# The influence of simultaneous lateral grafting on clinical outcomes following one-stage implant placement: a cross-sectional analysis

**DOI:** 10.1186/s40729-020-00226-6

**Published:** 2020-07-14

**Authors:** Ausra Ramanauskaite, Frank Schwarz, Amira Begic, Puria Parvini, Maria Elisa Galarraga-Vinueza, Karina Obreja

**Affiliations:** 1 Department of Oral Surgery and Implantology, Carolinum, Goethe University, Theodor-Stern-Kai 7, Building 29, 60596 Frankfurt am Main, Germany; 2 Department of Oral Surgery and Implantology, Carolinum, Johann Wolfgang Goethe-University Frankfurt, 60596 Frankfurt am Main, Germany; 3 Federal University of Santa Catarina (UFSC), Florianópolis, SC Brazil; 4 Department of Orthodontics, Westdeutsche Kieferklinik, Universitätsklinikum Düsseldorf, 40225 Düsseldorf, Germany

**Keywords:** Alveolar ridge augmentation, Guided bone regeneration, Dental implant

## Abstract

**Background:**

To investigate the influence of lateral bone augmentation procedures performed simultaneously with implant placement on peri-implant health or disease.

**Material and methods:**

A total of 232 patients showing the same type of a two-piece implant placed either simultaneously with lateral bone grafting using a bovine bone mineral and a native collagen membrane (*n* = 291 implants; test group) or at pristine bone sites without lateral bone grafting (*n* = 283 implants; control group) were enrolled in this cross-sectional analysis. Clinical outcomes (i.e., modified plaque index (mPI), bleeding on probing (BOP), probing depth (PD), and mucosal recession (MR)), and the frequency of peri-implant disease were evaluated after a mean follow-up period of 9.97 ± 6.55 years.

**Results:**

No differences were found between the patients in the test and control groups for any of the parameters investigated (i.e., mPI, BOP, PD, and MR). For the implants in both groups, PD values of 4–6 mm were more frequently noted in the upper jaw. A significant correlation between increased PD values and a larger implant diameter was noted for test implant sites. A KM of < 2 mm was associated with increased MR values in both groups. The prevalence of peri-implant mucositis and peri-implantitis was 68% and 5% for the patients in the test group and 61% and 10% in the control group, respectively.

**Conclusions:**

Simultaneous lateral grafting was associated with peri-implant tissue health and stability.

## Introduction

It is well documented that tooth extraction is followed by inevitable dimensional changes of the alveolar ridge, leading to reduced overall ridge volume and changes in the ridge shape [1]. As a consequence, once a dental implant—supported reconstruction—is chosen as a treatment option to fill the edentulous space, bone augmentation procedures are frequently required, either concomitant with implant placement or as a staged intervention.

In clinical situations involving horizontal alveolar ridge deficiencies, lateral bone augmentation procedures have been shown to effectively increase bone width, rendering implant placement in a second-stage surgery feasible [2]. Furthermore, procedures to regenerate the lateral alveolar ridge, when performed simultaneously with dental implant placement, have demonstrated a capacity to markedly reduce peri-implant bone defects, leading to a mean defect fill of 81.3% [3]. In fact, the optimal reduction of the defect height was more favorable when a combination of a grafting material and a barrier membrane was used, compared to a membrane or grafting material alone or the absence of treatment [3].

The potential influence of residual defects following lateral bone grafting has become a matter of concern, since it may be associated with an increased risk for peri-implant mucosal inflammation and subsequently progressive bone loss [4, 5]. Even though the results from a recent systematic review and meta-analysis have indicated that the changes in BOP over time were minimal [*n* = 10 studies; WMD = − 10.02%; 95% CI (− 22.23, 2.21)], it was also emphasized that the underlying evidence is weak. In fact, clinical and radiographic parameters to differentiate peri-implant health from disease have been inconsistently used in the evaluated studies and did not consider currently established case definitions for peri-implant disease [5].

Therefore, the aim of the present cross-sectional study was to further investigate the influence of lateral bone augmentation procedures performed simultaneously with implant placement on the maintenance of peri-implant health.

## Materials and methods

### Study design

The present cross-sectional analysis included 232 partially or fully edentulous patients (134 females and 98 males) exhibiting 574 implants (Ankylos®, DENTSPLY Implants Manufacturing GmbH, Mannheim, Germany). All implants were placed in the Department of Oral Surgery and Implantology, Goethe University, Frankfurt, following a standardized treatment protocol. Each patient had received a detailed description of the procedure, and an informed consent form was obtained prior to participation. The procedures in this study were in accordance with the Declaration of Helsinki, as revised in 2013, and the study protocol was approved by the local ethics committee (registration number 78/18).

### Patient selection criteria

The following inclusion criteria were applied for patient selection:

- Patients with > 18 years of age rehabilitated with at least one Ankylos® implant;
- Patients with treated chronic periodontitis and regular maintenance care;
- Non-smokers, smokers, and former smokers;
- A good level of oral hygiene as evidenced by a plaque index (PI) < 1 at the implant level;
- Attendance of a yearly routine implant maintenance appointment.

Patients were excluded for the following conditions: systemic diseases that could influence the outcome of the therapy, such as diabetes (HbA1c < 7), osteoporosis; a history of malignancy, radiotherapy, chemotherapy, immunodeficiency, or antiresorptive therapy; and pregnancy or lactation at the last follow-up.

### Treatment protocol

Two-piece platform-switched implants (Ankylos®, Dentsply Implants Manufacturing GmbH, Mannheim, Germany) were placed in a prosthetically ideal position and, according to the manufacturer’s surgical protocol, considering a subcrestal positioning of the implant shoulder. Implants in the control group displayed an intact vestibular alveolar bone wall without the need for a lateral bone grafting procedure. Implants in the test group exhibited dehiscence-type defects at the vestibular aspect, which were simultaneously filled with a particulated bovine bone mineral (Bio-Oss spongiosa granules sized 0.25–1 mm, Geistlich, Wolhusen, Switzerland) and covered by a native collagen membrane (Bio-Gide, Geistlich, Wolhusen, Switzerland). While control sites were left to heal in a transmucosal position, all test sites were submerged for a healing period of 4 to 6 months. The implants in both test and control groups were mainly restored with fixed cemented (control 273; test 264) crowns (control 188; test 190) and bridges (control 68; test 59). Screw-retained (control 9; test 25), telescopic (control 5; test 4), and removable restorations (control 0; test 22) were less frequent.

### Implant and implant-site characteristics

The following study variables were assessed for both test and control implants:

(1) Implant age (i.e., defined as time after implant placement), (2) implant location (i.e., upper or lower jaw, anterior (i.e., canine to canine) or posterior (i.e., premolar and molar region) segments), and (3) implant diameter.

### Clinical measurements

The following clinical parameters were registered at each implant site using a conventional periodontal probe:

(1) Modified plaque index (mPI) (Löe et al.) [6], (2) bleeding on probing (BOP)—measured as presence/absence, (3) probing depth (PD)—measured from the mucosal margin to the probable pocket, (4) mucosal recession (MR)—measured from the restoration margin to the mucosal margin, and (5) keratinized mucosa (KM) (mm).

Modified PI, BOP, PD, and MR measurements were performed at six sites per implant: mesiobuccal (mb), midbuccal (b), distobuccal (db), mesiooral (mo), midoral (o), and distooral (do). KM measurement was performed at three aspects per implant: mesiobuccal (mb), midbuccal (b), and distobuccal (db).

The presence of peri-implant disease at each implant site was assessed according to established case definitions [7]:

- Peri-implant mucositis defined as the presence of BOP and/or suppuration with on gentle probing with or without increased PDs compared to previous examinations, and an absence of bone loss beyond crestal bone level changes resulting from initial bone remodeling.
- Peri-implantitis defined as the presence of BOP and/or suppuration on gentle probing, increased PDs compared to previous examination, and the presence of bone loss beyond crestal bone level changes resulting from initial bone remodeling.

### Radiographic assessment

When clinical signs suggested the presence of peri-implant tissue inflammation, panoramic radiographs were assessed. To evaluate the bone-level changes at the implant sites, the obtained radiographs were compared with the baseline situation (i.e., radiographs taken following the placement of the final prosthetic reconstruction). After digitalization of the radiographs (Microtek ScanMaker i800 Plus, Hsinchu, Taiwan; LaserSoft Imaging AG, Kiel, Germany), measurements (i.e., bone-level changes between the baseline and follow-up radiographs) were performed employing the Sidexis XG software (Sirona Dental Systems GmbH, Bensheim, Germany). The measurement scale was based on the known implant height. Two reference horizontal lines were used: one marked the most coronal point of the peri-implant bone crest at mesial and distal sites (BC), and another traced the implant’s most apical point (AP). Vertical lines parallel to the reference line crossing the long axis of the implant were traced perpendicularly to the BC and AP at mesial and distal sites.

### Statistical analysis

The statistical analysis was performed using a commercially available software program (SPSSStatistics 26.0: IBM Corp., Ehningen, Germany). Descriptive statistics (means, standard deviations, medians, and 95% confidence intervals) were calculated for mPI, BOP, PD, and MR values. The analysis was performed at the patient and implant levels. The data were tested for normality by means of the Shapiro-Wilk test. Between-group comparisons of clinical outcomes were accomplished using the unpaired *t* test. Logistic regression analyses based on the implant-level data were used to depict relationships between BOP, PD, or MR values and the following variables: implant location (anterior/posterior), diameter (< 4.5/≥ 4.5 mm), and KM (< 2/≥ 2 mm). The alpha error was set at 0.05.

## Results

### Patient, implant, and implant site characteristics

The test group enrolled 64 women and 47 men with a total of 283 implants. The sample in the control group consisted of 70 women and 51 men with 291 implants. The mean estimated patient age in the test and control groups was 64.84 ± 12.15 and 65.19 ± 12.2 years, respectively.

The mean implant functioning time was 8.12 ± 5.12 years for the test group and 11.76 ± 7.19 years for the control group. Majority of the included implants had a diameter of 3.5 mm (test 77%; control 82%) with a mean facial KM width of 2.47 mm in the test group and of 2.84 mm in the control group (Table 1). In the test group, most implants were located in the posterior region of the lower jaw (44%), whereas the most common implant location in the control group was the posterior segment of the upper jaw (56%).

**Table 1:** Patient and implant site characteristics

	Control group (*n* = 283 implants)	Test group (*n* = 291 implants)
Patient number	*n* = 121	*n* = 111
Patient age	65.19 ± 12.2 years	64.84 ± 12.15 years
Patient gender (female/male)	70/51	64/47
Implant age years (mean ± SD)	11.76 ± 7.19 years	8.12 ± 5.21 years
Location upper jaw: anterior/posterior segment (*n*)	47/158	35/79
Location lower jaw: anterior/posterior segment (*n*)	16/62	48/129
Keratinized mucosa (mm)	2.84 ± 1.61	2.47 ± 1.52
Implant diameter: 3.5/4.5/5.5 mm (*n*)	233/48/2	225/61/5

### Clinical measurements

The clinical measurements are presented in Table 2. In general, all patients and implant sites exhibited low mPI scores (*patient-level:* test 0.49, control 0.46; *p* = 0.56: *implant-level:* test 0.51 and 0.49, respectively; *p* = 0.51). Mean BOP scores and MR values were comparable between the patients in the test and control groups (test 32.26% and 0.18 mm; control 33.07% and 0.17 mm, respectively), and similar at test and control implant sites (test 35.53%, 0.19 mm; control 36.77%, 0.16 mm, respectively). With regards to the mean PD scores, based on the patient-level data, no difference was noted between the two groups (test 2.86 mm: control 3.0 mm; *p* = 0.22), whereas implant-level estimations pointed toward higher mean PD values at the test implant sites (3.0 mm vs. 2.78 mm; *p* = 0.002).

**Table 2:** Clinical parameters (mean ± SD, median, and 95% CI)

Clinical parameters	Control group (mean ± SD)	Median	95% CI	Test group (mean ± SD)	Median	95% CI	***p***
**Modified plaque index**							
Patient-level	0.46 ± 0.43	0.33	0.38–0.54	0.49 ± 0.42	0.50	0.41–0.57	0.56
Implant-level	0.49 ± 0.41	0.33	0.44–0.54	0.51 ± 0.43	0.50	0.46–0.56	0.51
**Bleeding on probing (%)**							
Patient-level	33.07 ± 30.37	33	27.36–38.79	32.26 ± 30.88	17	26.71–37.82	0.084
Implant-level	36.77 ± 32.23	33	33–40.10	35.53 ± 32.67	33	31.76–39.30	0.648
**Probing depth (mm)**							
Patient-level	3.0 ± 0.72	2.83	2.86-3.13	2.86 ± 0.96	2.67	2.68-3.03	0.22
Implant-level	3.0 ± 0.85	2.83	2.90–3.11	2.78 ± 0.86	2.67	2.68–2.88	0.002
**Mucosal recession (mm)**							
Patient-level	0.17 ± 0.34	0	0.10–0.23	0.18 ± 0.36	0	0.11–0.24	0.79
Implant-level	0.16 ± 0.36	0	0.12–0.20	0.19 ± 0.36	0	0.16–0.24	0.221

### Incidence of peri-implant disease

The frequency distribution of peri-implant disease in the test and control groups is summarized in Table 3. According to the given case definitions, 68% of the patients in the test group and 61% of the patients in the control group were diagnosed with peri-implant mucositis, while peri-implantitis was diagnosed in 5% of the patients in the test and in 10% of the patients in the control group. At the implant level, the corresponding values amounted to 66% and 5% in the test group and 64% and 8% in the control group, respectively (Table 3).

**Table 3:** Prevalence of peri-implant health and disease

	Patient-level control group	Test group	Implant-level control group	Test group
Healthy	32	33	79	83
Peri-implant mucositis	68	82	181	192
Peri-implantitis	11	6	23	16

### Regression analysis

Cross-tabulations between selected independent variables (PD, MR, and BOP values) and local factors (i.e., implant region, implant diameter, and KM) in the test and control groups are summarized in Tables 4 and 5.

**Table 4:** Test group (*n* = 291 implants) cross-tabulations: a) BOP/PD/MR values and (1) implant region, (2) implant diameter, and (3) KM

**(1) BOP values**	**Implant region**		
	Upper jaw	Lower jaw	
**0**	33	50	
**< 33%**	22	40	
**< 67%**	31	47	
**> 67%**	28	40	
	**Implant diameter**		
	3.5 mm	4.5 mm	5.5 mm
**0**	67	14	2
**< 33%**	47	15	0
**< 67%**	63	14	1
**> 67%**	48	18	2
	**KM**		
	< 2 mm	≥ 2 mm	
**0**	24	59	
**< 33%**	21	41	
**< 67%**	17	61	
**> 67%**	23	45	
**(2) PD values**	**Implant Region**		
	Upper jaw	Lower jaw	
**1–3 mm**	74	161	
**4–6 mm**	40	16	
**> 7 mm**	0	0	
	**Implant diameter**		
	3.5 mm	4.5 mm	5.5 mm
**1–3 mm**	188	45	2
**4**–**6 mm**	37	16	3
**> 7 mm**	0	0	0
	**KM**		
	< 2 mm	≥ 2 mm	
**1-3 mm**	70	165	
**4-6 mm**	15	41	
**> 7 mm**	0	0	
**(3) MR**	**Implant region**		
	Upper jaw	Lower jaw	
**0 mm**	72	119	
**> 0 mm**	42	58	
	**Implant diameter**		
	3.5 mm	4.5 mm	5.5 mm
**0 mm**	145	42	4
**> 0 mm**	80	19	1
	**KM**		
	< 2 mm	> 2 mm	
**0 mm**	43	148	
**> 0 mm**	42	58	

*BOP* bleeding on probing, *PD* probing depth, *MR* mucosal recession, *KM* keratinized mucosa

**Table 5:** Control group (*n* = 283 implants) cross-tabulations: a) OP/PD/MR values and (1) implant region, (2) implant diameter, and (3) KM

**1) BOP values**	**Implant region**		
	Upper jaw	Lower jaw	
**0**	59	20	
**< 33%**	33	13	
**< 67%**	56	26	
**> 67%**	57	19	
	**Implant diameter**		
	3.5 mm	4.5 mm	5.5 mm
**0**	64	15	0
**< 33%**	35	11	0
**< 67%**	69	12	1
**> 67%**	65	10	1
	**KM**		
	< 2 mm	> 2 mm	
0	12	67	
< 33%	12	34	
< 67%	19	63	
> 67%	22	54	
**(2) PD values**	**Implant region**		
	Upper jaw	Lower jaw	
**1–3 mm**	138	70	
**4–6 mm**	64	8	
**> 7 mm**	3	0	
	**Implant diameter**		
	3. 5 mm	4.5 mm	5.5 mm
**1**–**3 mm**	174	33	1
**4**–**6 mm**	57	14	1
**> 7 mm**	2	1	0
	**KM**		
	< 2 mm	> 2 mm	
**1**–**3 mm**	41	167	
**4**–**6 mm**	23	49	
**> 7 mm**	1	2	
**(3) MR**	**Implant region**		
	Upper jaw	Lower jaw	
**0 mm**	153	55	
**> 0 mm**	52	23	
	**Implant diameter**		
	3.5 mm	4.5 mm	5.5 mm
**0 mm**	172	34	2
**> 0 mm**	61	14	0
	**KM**		
	< 2 mm	> 2 mm	
**0 mm**	29	179	
**> 0 mm**	36	39	

*BOP* bleeding on probing, *PD* probing depth, *MR* mucosal recession, *KM* keratinized mucosa

In both groups, implants located in the upper jaws were more frequently associated with PD values of 4–6 mm than in the lower jaws (*p* = 0.001, respectively). In the test group, PD values were commonly higher at implants exhibiting larger diameters (i.e., 4.5 and 5.5 mm vs. 3.5 mm; *p* = 0.04). For control group implants, BOP scores of > 67% and PD values of 4–6 mm were more frequently noted at implant sites exhibiting KM < 2 mm (*p* = 0.03 and *p* = 0.017, respectively). Moreover, at both test and control implants, KM values of < 2 mm were correlated with increases in MR valuers (*p* = 0.001, respectively).

## Discussion

The present cross-sectional study aimed at evaluating the influence of lateral bone augmentation procedures performed simultaneously with implant placement on peri-implant health or disease.

Based on the present data, none of the investigated clinical parameters (i.e., mPI, BOP, PD, and MR) differed between the patients treated with simultaneous lateral bone augmentation and those treated with implants placed in pristine bone without hard tissue grafting. The implant-level analysis, however, pointed toward higher mean PD values for the grafted implant sites (3.0 vs. 2.78 mm). Our findings basically corroborate the results of one previous randomized clinical trial that elaborated upon the effects of lateral bone grafting around dental implants presenting with dehiscence-type defects (≤ 5 mm) at the vestibular aspect over a spontaneous defect healing (i.e., without grafting) [8, 9]. In particular, a more pronounced vertical bone loss during the first 6 months following implant placement as well as significantly higher residual vertical dehiscence defects was noted following spontaneous healing, as compared to the grafted implant sites (− 0.17 mm and 2.51 vs. 1.79 mm and 1.61, respectively) [8, 9]. Nevertheless, the clinical performance of the implants in the test and control groups was similar, and peri-implant tissue health with low mucosal inflammatory (i.e., sulcus bleeding index) changes was maintained over the mean investigation period of 7.5 years [8].

Opposing clinical data, on the other hand, have demonstrated that residual dehiscence-type defects and subsequently exposed rough implant surfaces negatively influenced peri-implant tissue health [10]. In particular, after 4 years, implant sites exhibiting advanced residual dehiscence-type defects (> 1 mm) on the vestibular aspect following simultaneous lateral grafting (natural bone mineral + native or cross-linked collagen membranes) revealed an increased risk of developing peri-implant tissue inflammation, thus emphasizing the need for optimizing defect fill when performing bone grafting simultaneous with implant placement [10]. Further results from the aforementioned investigation showed a tendency toward higher MR values, especially on the midbuccal aspect of implant sites exhibiting residual defects of > 1 mm [10]. The latter observation is in accordance with the results of a recent cross-sectional analysis, which detected a more apical position of the first bone-to-implant contact at the vestibular aspect for implants presenting with mucosal recessions (4.85 mm vs. 2.15 mm, respectively) [11]. Similar findings were also observed at immediately placed implants, since implant sites showing no radiographic bone at the buccal site were associated with an apical shift (1 mm) of the mucosal level when compared with implants showing an intact buccal bone [12]. In the present analysis, the overall mean MR values were low and similar at both test and control implant sites (0.18 mm and 0.17 mm, respectively). This is in disagreement with the previous data suggesting more favorable mucosal levels at implant sites treated without GBR than at sites that underwent lateral GBR [9]. In this context, it must be emphasized that the buccal bone levels were not evaluated in the present study and therefore, the potential presence of residual defects in the test group could not be assessed. Apart from that, peri-implant tissue health and esthetics appeared to not be jeopardized by either thin or missing vestibular bone at the implants in the maxillary anterior region [13]. One might speculate that those findings, to some certain extent, could be explained by the inverse relationship noted between bone and soft-tissue thickness [14]. In particular, one pre-clinical study employing a canine model observed a physiological increase in the peri-implant mucosa thickness as a compensation for underlying vestibular bone deficiencies, with the highest horizontal mucosa thickness detected in the absence of vestibular bone plate [14].

When further interpreting the results of the present study, the regression analysis for the test implant sites revealed a significant association between larger implant diameter (i.e., 4.5–5.5 mm vs. 3.5 mm) and increased PD values (i.e., 4–6 mm). This finding might be partially attributable to the fact that inserting a larger implant diameter may lead to an implant location being outside of the bony envelope, resulting in uncontained peri-implant defects. From a biological perspective, such defects feature a compromised regenerative potential; thus, the presence of increased PDs may be a clinical sign pointing to an incomplete defect regeneration.

Regression analysis also demonstrated that control implant sites with reduced KM values (< 2 mm) were frequently associated with profuse mucosal bleeding ( 67%) and increased PD values of 4–6 mm compared to the implants exhibiting KM ≥ 2 mm. This tendency may be related to the fact that reduced KM width (< 2 mm) was shown to negatively affect self-performed oral hygiene measures, which subsequently increased the likelihood of peri-implant mucosal inflammation [15]. Another noteworthy finding of the present study was the increased risk for mucosal recession at implants exhibiting KM of < 2 mm. This tendency aligns with the results of one previous cross-sectional analysis, which found KM of > 2 mm being a protective factor against peri-implant soft-tissue dehiscence, whereas a more apical soft-tissue position on the vestibular aspect was frequently detected at the implant sites with KM < 2 mm [11].

The present prevalence of peri-implant mucositis and peri-implantitis between the test and control groups was similar, amounting to 66% and 5% in the implant test group and 64% and 8% in the control group, respectively. A slightly higher frequency of peri-implant mucositis was indicated in a recent clinical trial, where 81% of the implants in the test group (i.e., dehiscence defects treated with lateral hard tissue grafting) and 79% in the control group (i.e., spontaneous defect healing) presented with bleeding upon probing procedure [8]. Note that in contrast to the results of the present study, none of the implants developed signs of peri-implantitis over the investigation period of 7.5 years [8]. The existing contradicting data, however, pointed toward a link between bone augmentation procedures and increased peri-implantitis risk (OR = 2) [16]. Moreover, patients exhibiting implants placed along with the bone grafting procedures had more than double the frequency of peri-implantitis (defined as BOP+ and/or suppuration, PD ≥ 4 mm, radiographic bone level > 3), as compared to those having implants inserted into the pristine bone (18% vs. 7%) [17].

Within the limitations of the present study, it was concluded that simultaneous lateral grafting was associated with peri-implant tissue health and stability.

## Data Availability

Not applicable.
